# Malignant subdural effusion associated with disseminated adenocarcinoma: a case report

**DOI:** 10.1186/1757-1626-1-328

**Published:** 2008-11-18

**Authors:** Seyed Mohammad Haji Mirsadeghi, Zohreh Habibi, Keyvan Tayebi Meybodi, Farideh Nejat, Seyed Ali Fakhr Tabatabai

**Affiliations:** 1 Department of Neurosurgery, Imam Khomeini Hospital, Tehran University of Medical Sciences, Tehran, Iran; 2 Departments of Neurosurgery, Children's Hospital Medical Center, Tehran University of Medical Sciences, Tehran, Iran

## Abstract

**Background:**

Subdural effusion in the setting of dural metastasis is very rare and may be difficult to be distinguished from chronic subdural hematoma.

**Case presentation:**

A 44-year old woman with gastric adenocarcinoma was presented with headache and a hypodense subdural collection in right fronto-parietal in brain CT. Burr-hole irrigation was performed with the impression of chronic subdural hematoma, but nonhemorrhagic xantochromic fluid was evacuated without malignant cell. Brain CT on the 11^th ^day depicted fluid re-accumulation and noticeable midline shift, necessitating craniotomy and removing the affected dura.

**Conclusion:**

Because the affected dura can be supposed as the main source of subdural effusion, resection of the involved dura is obligatory for the appropriate palliative management of such patients.

## Background

Subdural effusion may occur following different situations such as bacterial meningitis, head injury and certain surgical procedures such as transventricular approaches or endoscopic third ventriculostomy [1-4]. However, subdural effusion in the setting of dural metastasis is very rare and to our knowledge limited cases have been reported [5].

In this report, a case of advanced gastric adenocarcinoma with systemic dissemination and metastatic subdural effusion is presented and different aspects regarding diagnosis, treatment and pathophysiology of the lesion, and differentiation from CSH are discussed.

## Case presentation

**Figure 1:**
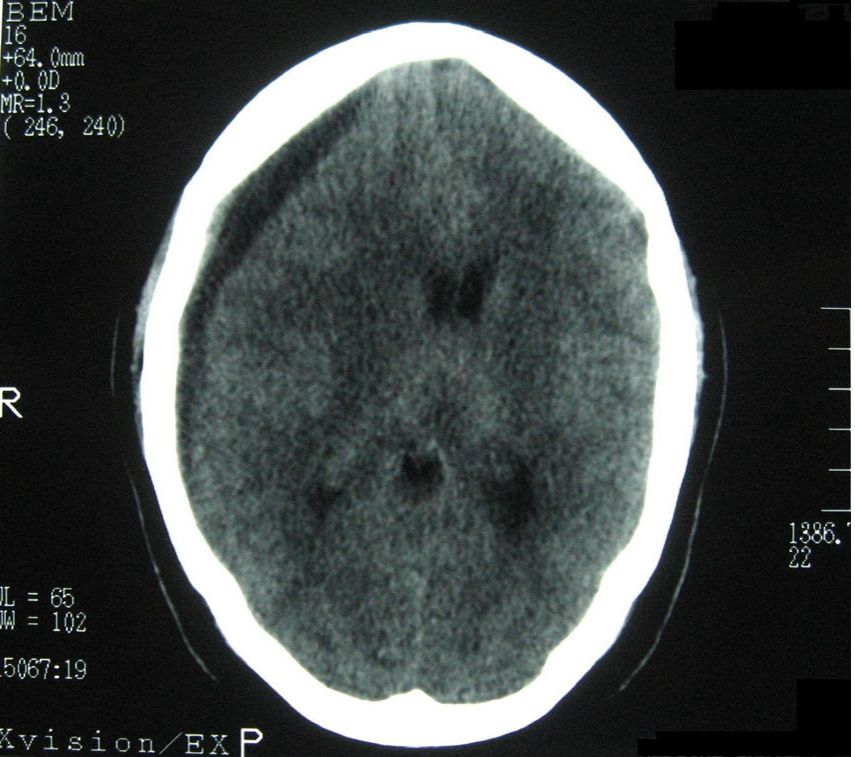
Noncontrast brain computed tomography showing a crescent-like hypodense area in the right fronto-parietal region with a tiny hyperdense rim in the lateral border of the lesion.

A 44-year old woman with a 2-year history of gastric adenocarcinoma and regional lymph nodes involvement was admitted with severe headache, drowsiness, and mild left hemiparesis. She had recently been diagnosed with multiple systemic metastases including bilateral ovarian masses suggestive for Krukenberg tumor, pulmonary lymphangitic carcinomatosis, and multifocal bone involvements. The patient received 3 courses of systemic chemotherapy before the onset of headache. She had neither history of prior head injury, nor anticoagulant consumption. On admission, the patient was conscious but agitated, and the force of the left extremities was of 4/5 according to Louisiana State University Medical Center grading system. Brain computed tomography (CT) revealed a crescent-like hypodense subdural collection in the right fronto-parietal region, 1 centimeter in diameter with a considerable midline shift to the left (Figure 1). A tiny hyperdense rim, approximately 2 mm in width and 1 cm in length, was observed in the lateral border of the lesion. Burr-hole irrigation was performed with the impression of chronic subdural hematoma (CSH) as a result of potential transient thrombocytopenia during the previous chemotherapies. Afterward, nonhemorrhagic xantochromic fluid was evacuated from subdural space. The evacuated fluid analysis revealed a high level of protein without any malignant cell. The patient's symptoms were dramatically improved after operation, and she discharged with good general condition 4 days later.

**Figure 2:**
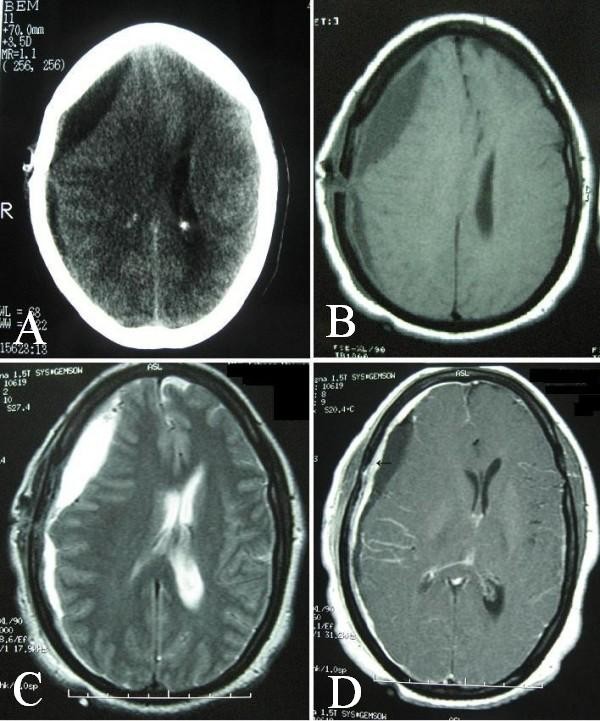
**Computed tomography obtained 11 days after surgery showing reaccumulation of the subdural effusion** (A). Magnetic resonance images showing right frontoparietal subdural fluid, hypointense in T1-weighted (B) and hyperintense in T2-weighted axial plane (C). Thick dural enhancement (arrow) and a pattern of gyral enhancement at the right parietal region (D).

Eleven days after surgery, the patient came back with more severe headache and subgaleal collection at the site of previous burr hole. New brain CT scan depicted re-accumulation of the subdural effusion and a noticeable right to left shift (Figure 2). The previous hyperdense rim was still present and even seemed to increase. Brain magnetic resonance imaging (MRI) with and without gadolinium injection displayed the right side subdural effusion, hypointense in T1-weighted and hyperintense in T2-weighted images, accompanied with an abnormal dural enhancement in the right parietal and a pattern of gyral enhancement at the same region (Figure 2).

Because of developing dysphasia and worsened hemiparesis during the assessments, re-operation was contemplated and right fronto-parietal craniotomy was performed. A gray-reddish thickened and hyperemic dura was disclosed. The affected dura was resected, and the borders of the dura in the vicinity of the involved region were coagulated carefully.

**Figure 3:**
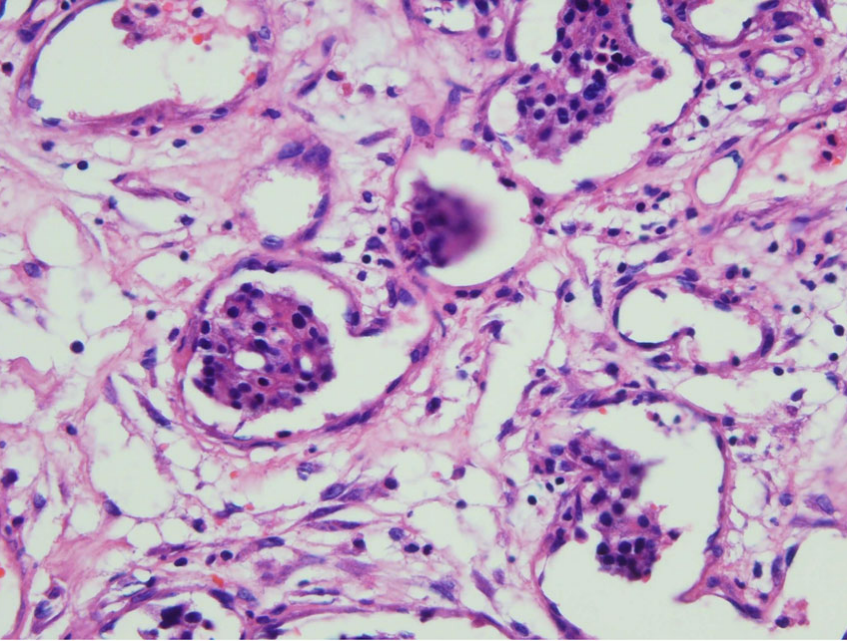
**Photomicrograph of the lesion showing thickened dura containing the nests of metastatic adenocarcinomatous and inflammatory cells.** H & E, original magnification ×100.

Histopathological examination revealed a thickened dura containing the nests of metastatic adenocarcinomatous and inflammatory cells (Figure 3).

The patient's headache and other symptoms partially improved post-operatively. During 5 months follow up, serial images showed no evidence of recurrent effusion, nor did the patient experience any other episode of headache. However, the systemic metastatic disease progressed continuously, and the patient died from pulmonary insufficiency.

## Discussion

Dural metastases account for about 3–13% of all intracranial metastatic involvements [6], with the condition being found in 8–9% of post mortem evaluation of advanced systemic cancers. The most common neoplasms associated with dural metastases are breast cancer, melanoma, gastrointestinal and prostate cancer [7]. The lesions often remain clinically asymptomatic [8], but they may become symptomatic due to mass effect or developing different kinds of subdural fluid from acute or chronic hematoma to malignant effusion [5,9,10]. Chronic subdural hematoma resulting from neoplastic invasion of the meninges is uncommon, with the incidence being about 0.5 – 4% of all intracranial metastases [6]. Metastaic subdural effusion is even scarcer, and to our best knowledge few cases of such condition have been reported beforehand [5,10].

### Diagnostic options

The differentiation between CSH and subdural effusion due to metastasis in CT scan images is difficult, as the subdural collection may mask the underlying dural involvement [6]. Based on the previous reports, the dural metastasis may have been diagnosed during the second surgery for re-accumulated effusion or hematoma [6,7,10]. Since subdural effusion is in the list of differential diagnosis of CSH, more accurate modalities such as MRI can be useful in patients with malignancy and subdural collection to provide a better distinction between non-hemorrhagic fluid and hemorrhagic products. In the present case, regarding the low signal of the collected fluid in T1-weighted and high signal in T-2 weighted images, compatible with cerebrospinal fluid attenuation, the lesion was considered to be effusion rather than hematoma. A better delineation of the probable underlying dural metastasis can be provided by MRI as well. However, MRI would be misleading when the metastasis simulates a meningioma or when the subdural fluid hides the mass [8]. For accurate diagnosis, biopsy of the involved dura and cytological examination is mandatory [6,7]. Nevertheless, failure to demonstrate neoplastic infiltration dose not necessarily exclude dural metastasis [6]. Moreover, in the most reported cases, no assistance from cytological examination has been revealed [6,10].

### Treatment

Considering the poor prognosis due to tumor dissemination in most cases, the patients usually undergo palliative treatment. Bur hole irrigation alone is inadequate and may lead to fluid re-accumulation necessitating craniotomy to evacuate the subdural fluid and dura exploration for removing the metastatic tumor [10]. Post-operative irradiation seems to be essential to control the residual neoplastic cells, although the prognosis is poor due to malignant dissemination.

### Hypotheses on etiology

Three theories can be postulated concerning the mechanism of subdural fluid accumulation in this case:

1- The fluid extravasation into subdural space may be caused by increased hydrostatic pressure which can be explained by venous or arterial mechanisms. Obstruction of the dural veins by tumor cells would cause dilation of the capillaries in the inner areolar layer [9,10], followed by increase in capillary transmural pressure and fluid extravasation into subdural space. Moreover, Fukino et al, have shown an increased cerebral blood flow (CBF) in the hemisphere with malignant subdural hematoma [9]. This pattern of altered CBF may exacerbate the fluid collecting process as well.

2- It has been shown that hyperplasia of the inner areolar layer occurs as an angiodesmoplastic response to the tumor invasion [9]. The fluid may be transudated from the immature angiogenesis.

3- Considering the elevated protein content and negative cytology of the fluid, it can be suggested that the mucine producing adenocarcinomatous cells may have potential secretory capability themselves, and the fluid would be originated directly from metastatic malignant cells.

In accordance with all mentioned mechanisms, the involved dura can be supposed as the main source of creating subdural effusion. Consequently, resection of the affected dura can control the effusion.

## Conclusion

In conclusion, dural metastasis should be considered in patients with malignant neoplasm and subdural collection. Because the affected dura itself is presumably responsible for fluid production, resection of the involved dura is obligatory for the appropriate palliative management of the metastatic dural effusion.

## Consent

Written informed consent was obtained from the patient's children for publication of this case report and accompanying images after her death. A copy of the written consent is available for review by the Editor-in-Chief of this journal.

## Competing interests

The authors declare that they have no competing interests.

## Authors' contributions

SMHM made contribution to conception and analyzed the patient data. ZH made contribution in collecting data and drafting the manuscript. KTM was contributor in drafting the manuscript. FN was contributor in revising the manuscript critically. SAFT has given the final approval for the version to be submitted.
